# The influence of previous arthroscopic treatment on subsequent primary total knee arthroplasty: the comparison between bilateral knees of the same patient

**DOI:** 10.1186/s12891-021-04003-3

**Published:** 2021-01-29

**Authors:** Kuishuai Xu, Liang Zhang, Rui Shen, Cailin Wang, Tianyu Li, Xia Zhao, Tengbo Yu

**Affiliations:** 1 Department of Orthopedics, The Affiliated Hospital of Qingdao University, Qingdao, 266000 Shandong China; 2 Department of Abdominal ultrasound, The Affiliated Hospital of Qingdao University, Qingdao, 266000 Shandong China; 3 Wenzhou Medical University, Wenzhou, 32500 Zhejiang China

**Keywords:** Influence, Arthroscopy, Total knee arthroplasty, Same patient

## Abstract

**Background:**

To explore whether previous arthroscopic knee surgery affects future total knee arthroplasty (TKA) results or not**.**

**Methods:**

A total of 56 patients with the previous arthroscopic treatment on one knee underwent subsequent bilateral total knee arthroplasty in our hospital from September 2012 to July 2018. Data on each patient were collected in regards to changes in postoperative clinical and functional scores, various other scores, as well as postoperative functional recovery and complications. We defined the knees with a previous arthroscopic history as group A, and the counter side as group B. The Knee Society clinical score, functional scores, range of motion (ROM), finger joint size (FJS), visual analogue scale (VAS) scores were assessed before and after surgery. Using the Kolmogorov-Smirnov Test to test the normality of continuous variables, and the chi-square test to compare the rate of reoperation and complications between two groups. For all statistical comparisons, *P* < 0.05 was considered significant.

**Results:**

There were no statistically significance differences found in postoperative Knee Society clinical scores and functional scores between group A and group B, as well as in ROM, FJS, VAS scores and local complications**.**

**Conclusion:**

There were no statistically significant differences found in postoperative functional recovery and complications in patients, who underwent total knee arthroplasty with previous knee arthroscopy.

## Background

The global burden of disease 2010 study showed that the global morbidity of symptomatic knee osteoarthritis was estimated to be 3.8%, and that the morbidity increases with age, and peaks at 50 years old [1]. TKA is currently proved to be an effective surgical method for patients with moderate or severe staged knee osteoarthritis and clinical practice has shown that it can significantly relieve the pain, improve the range of motion, increase the quality of life [2, 3]. However, adverse events after TKA remain a severe challenge for surgeons, Postoperative complications such as venous thromboembolism (VTE), infection, periprosthetic fracture and dislocation have occurred in some patients [4, 5]. A study recommended that use of arthroscopic debridement is a feasible method for osteoarthritis [6]. Several studies have evaluated the influence of TKA in patients with prior knee arthroscopy in recent years, but the results remain controversial [7–10]. Some studies suggested that prior arthroscopy resulted in longer operative time, higher rates of postoperative complications and reoperation, as well as a shorter implant working life. Other studies argued that prior arthroscopy has a negative impact on subsequent TKA [9].

In general, we still determine whether previous arthroscopy procedures can influence the clinical effects of subsequent future TKA and whether the surgeon and patients should pay more attention to previous procedures. Mutual controls were adopted by most previous studies to eliminate the impact of demographic variables—age, sex, body mass index between control groups and study groups. Nevertheless, some factors are hard to control and quantify, such as lifestyle, conditions of limbs exercise and rehabilitation. In this study, we selected the same patient to improve the study design. The clinical curative effects evaluation and the rate of complications were compared in both knees to evaluate the influence of prior arthroscopy on following TKA.

## Methods

From September 2012 to July 2018, According to inclusion and exclusion criteria,56 patients fit the criteria for bilateral knee arthroplasty following knee arthroscopy. All Patients were followed for at least 24 months.

### Inclusion and exclusion criteria

The inclusion criteria were: 1) patients who underwent simultaneously or staged TKA from September 2012 to July 2018. 2) patients with a history of unilateral arthroscopy. 3) patients with complete follow-up data. 4) Patients used the same type of knee prosthesis on both sides of the knee.

The exclusion criteria were: 1) patients with prior a cruciate ligament reconstruction history. 2) patients who underwent knee revision. 3) patients who underwent TKA combined further operations under single anesthesia. 4) patients with infectious osteoarthritis, knee fracture or other knee operation.

56 patients met the inclusion and exclusion criteria, including 7 males and 49 females. The mean age at the time of TKA was 62.89 ± 6.30 years (46–73) in the study group and 63.06 ± 6.27 years (47–74) in the control group. The mean age, mean body mass index and ASA had no statistical significance between both groups.

### General information

From September 2012 to July 2018, 483 patients underwent TKA following arthroscopy in our hospital. Patients who underwent unilateral knee arthroplasty or patients with a history of bilateral knee arthroscopy were excluded, and finally, 56 patients (112 knees) were composite inclusion criteria. Among the 56 patients, 13 patients underwent simultaneous TKA and the other patients underwent staged TKA——7 patients were performed staged TKA within 1 month, 5 patients were more than 1 months but less than 12 months, and others were more than 12 months. We defined the knee with a prior arthroscopy history as group A and the other side as group B. All patients were followed up to 1 September 2020.

### Therapeutic process

All the operations were performed by experienced surgeons in our hospital. Both knees were also completed by the same surgeon. The medial parapatellar approach was used in all patients. 30 patients were implanted with Medial pivot (MP) prosthesis while 26 patients were implanted with Posterior-Stabilized (PS) knee prosthesis, all the patients did not give receive a replacement of patella, but we rubbed abraded it down to gain a better motion curve. Negative pressure drainage tubes were placed on each side knee in every patient after the operation, and all drainage tubes were removed within 24 h. Half an hour before the operation, the intravenous of traumatic acid (TXA) was chosen to prevent bleeding. We selected third-generation antibiotic prophylaxis to prevent infection and low molecular weight heparin as an anti-coagulent. When the drainage tubes were removed, all patients received daily rehabilitation training with the help of a professional therapist.

### Assessment

The database that contains essential information laboratory examination imaging examination information of surgery and clinical records was obtained from electronic medical record systems in our hospital. Indexes were assessed with postoperative local complications and functional scores. Local complications include infection, pathological dislocation, periprosthetic fracture, aseptic loosening, ankylosis and wound complication. Functional evaluations included ROM Knee Society score and FJS score. Besides, VAS was used for patient satisfaction. Comparisons of the aforesaid indexes between Group A and Group B were performed to evaluate the influence of the prior arthroscopy history on TKA.

### Statistical analysis

All statistical testing methods used were performed using SPSS 25 software in this study. As for continuous variables (ROM/KSS/FJS), the Kolmogorov-Smirnov test was first applied to test normality. The data are reported using descriptive statistics such as mean ± standard deviation or median ± quartile interval for continuous variables or frequencies for categorical variables. Statistical comparisons of preoperative and postoperative ROM/KSS/FJS were completed with the paired t-test or Wilcoxon Pearson rank-sum test as well as measurement data between group A and group B, and the chi-square test was applied to compare the rate of reoperation and complication between two groups. For all statistical comparisons, *P* < 0.05 was considered significant.

## Results

The mean follow-up time of the study group was slightly longer than that of the control group (43 months vs 41 months), but there was no statistical significance. The mean follow-up time was 58.12 ± 9.73 months (26–77). All basic information is presented in Table 1.

**Table 1:** Demographics and comorbidity profile of the patients

	Experimental group	Control group	P
Age (years)	62.89 ± 6.30 (46–73)	63.06 ± 6.27 (47–74)	0.160
Gender (male/female)	7/49	7/49	–
BMI (kg/m^2^)	27.96 ± 2.97 (21.48–35.54)	27.79 ± 3.01 (22.03–35.54)	0.541
**ASA**			0.813
1	1	1	
2	29	28	
3	26	27	
**Anesthesia**			0.810
General anesthesia	31	32	
Non-general anesthesia	25	24	
**Comorbidity**			
Hypertension	22	22	1.000
Coronary heart disease	8	8	1.000
Diabetes	14	14	1.000
Cerebral infarction	1	1	1.000
Respiratory system	6	6	1.000
Digestive system	2	2	1.000
Urinary system	1	1	1.000
**K-L grading**			
III	18	25	0.174
IV	38	31	0.174

A comparison of preoperative and postoperative KSS score between groups are shown in Table 2, including clinical score and functional score. There was no statistical significance in postoperative Knee Society clinical score and functional score between group A and group B (*P* > 0.05).

**Table 2:** Clinical efficacy score

	KS-C			KS-F		
	Pre-operation	Last follow up	P	Pre-operation	Last follow up	P
Group A	33.58 ± 2.63	83.58 ± 6.54	0.000	30.00 ± 4.14	81.53 ± 6.19	0.000
Group B	34.19 ± 2.93	84.67 ± 5.88	0.000	30.28 ± 3.15	82.08 ± 5.65	0.000
P	0.339	0.377		0.757	0.657	

ROM, FJS and VAS scores are shown in Table 3. There was no statistical significance in the preoperative ROM score, and the postoperative ROM score was also equivalent between groups (107.92 ± 10.98 ml versus 108.75 ± 10.98 ml, *P* = 0.638) at the latest follow-up. Similarly, the statistical analysis of FJS and VAS in both study groups showed no statistical significance.

**Table 3:** ROM/FJS/VAS

	ROM			FJS	VAS
	Pre-operation	Last follow up	P		
Group A	98.06 ± 16.18	107.92 ± 10.98	0.000	64.94 ± 9.86	7.47 ± 1.34
Group B	96.25 ± 16.45	108.75 ± 10.98	0.000	63.89 ± 7.59	7.58 ± 1.20
P	0.378	0.638		0.521	0.706

During the follow-up period, we identified two local complications (3.6%) in the study group, including one periprosthetic joint infection and one poor healing of the incision. We found that there were three complications (5.4%) in the control group, including two periprosthetic joint infection and one poor healing of the incision. None of the patients experienced aseptic loosening, dislocation, periprosthetic fracture or manipulations under anesthesia for stiffness. Although the complication rate after TKA was slightly higher in group B, there was no statistical significance in two groups (5.4% versus 3.6%, *P* > 0.05). The complications are shown in Table 4.

**Table 4:** Local complications

	Group A (*n* = 56)	Group B(*n* = 56)	P
Complications			
Infection	1	2	1.000
Poor healing of the incisn	1	1	1.000
Aseptic loosening	0	0	–
Dislocation	0	0	–
Periprosthetic fracturs	0	0	–
Stiffness	0	0	–
Revision for any reason	1	2	1.000

Three patients had revision surgery because of infection, one in Group A and the others in Group B. One patient in group A required revision surgery at 4 months postoperatively with the culture results indicating *Staphylococcus aureus* growth. The two patients in group B required the operation, one at 8 months postoperatively and one at twenty-four months after operation. One culture result indicating *Staphylococcus aureus* growth and the other culture results indicating staphylococcus epidermis growth. The rate of revision in Group A was slightly higher than Group B, but it was not statistically significant between the two groups (5.6% versus 2.8%, *P* = 1.000).

## Discussion

Only a few studies have paid attention to the influence of prior knee operation on subsequent TKA, including arthroscopy [7–10], ACL reconstruction [11, 12], bone and soft tissue knee surgery [13, 14], but the outcomes were conflicted. In this study, we compared bilateral knees in the same patient, when it comes to the local complication rates and clinical curative effects evaluation (FJS, KSS, ROM, VAS), we hold that there was no statistical significance. To our knowledge, it is the first comparative study in the same patient to examine the influence of prior arthroscopy on subsequent TKA.

Among these prior studies, only 4 previous studies evaluated the influence of prior arthroscopy on subsequent TKA [7–10]. In 2009, Piedade [6] reported the influence on TKA following prior arthroscopy for the first time. In their study, 60 patients who underwent TKA with previous arthroscopic history were included as a study group and 1119 patients without knee surgery as a control group. Statistical analysis revealed that the study group had a higher postoperative complication rate and a lower working rate of the prosthesis. However, statistical analysis did not reveal a statistical significance in postoperative function and pain scores between two groups.

Then Werner and Barton [8, 10] studied the influence of the interval time between arthroscopy history and following TKA. Werner divided 3051 patients with previous knee arthroscopy into three separate cohorts according to the interval time: TKA within 6 months after knee arthroscopy (*n* = 681), TKA between 6 months and 1 year after knee arthroscopy (*n* = 1301) and TKA from 1 to 2 years after knee arthroscopy (*n* = 1069). 37,235 TKA patients without previous knee arthroscopy were created as the control group. The authors found the incidences of infection (OR 2.0, *P* = 0.004), stiffness (OR 2.0, *P* = 0.001) and VTE (OR 1.6, *P* = 0.047) were higher in patients who underwent TKA within 6 months after knee arthroscopy compared to the patients in the control group. They also found that there was no increase in complications when TKA was performed more than 6 months after knee arthroscopy.

Similarly, Barton found that the interval time was a crucial factor for the function of patients who performed TKA with a prior arthroscopy history. Patients who performed TKA within 6 months of prior arthroscopy had a significant reduction in OKS. However, Anthony [8] conducted the study for a long term with average follow-up time up to 9 years, and found there was no statistical significance in KSS, ROM, complication rates and the working time of prosthesis between both groups.

However, the above studies were all mutual control experiments from one patient to another patient. Although confounding variables, such as general condition operative procedures,and rehabilitation during hospitalization, were mitigated through the matched cohort, the impact of postoperative factors, such as lifestyle medication were hard to control. Compared to these studies, the strength of this particular study is self-controlled design. Patients who underwent simultaneously or staged TKA with a history of arthroscopy in one knee were included and the outcomes were compared in bilateral knees within the same patient. However, TKA performed by different surgical teams, using different prostheses or other different surgical techniques, were excluded. Therefore, this study can not only eliminate the discrepancy involving baseline, operative procedures, rehabilitation during hospitalization but also eliminate the impact of the postoperative lifestyle, the work conditions, use of medication and exercise conditions. This research method has already been applied to compare AS prosthesis and PS prosthesis [15], Computer-Navigated TKA and Conventional TKA [16], highly cross-linked prosthesis and conventional prosthesis [17], UKA and TKA [18, 19]. However, to our knowledge, it has not been adopted to evaluate the influence of prior arthroscopy on subsequent TKA.

At the latest follow-up, this study showed no statistical significance for KSS, FJS, ROM and VAS between two groups to previous reports. That is to say,the prior arthroscopy does not influence functional score and patient’s satisfaction. Anthony [9] identified 480 patients (160 patients as arthroscopy group, 320 patients matched 2:1 as controls) and found no statistical significance in KSS score and ROM between two groups for ten years of follow-up. Piedade [7] identified 60 primary TKA with previous arthroscopic debridement as a study group and 1119 primary TKA without surgery as a control group. Statistical analysis of postoperative International Knee Society (IKS) and ROM showed no difference.

In our study, the complication rate was found to be equivalent between the two groups, which are consistent with most previous reports. In the 2:1 matched control study, Anthony [9] found the curatorship free of complication at 5 years and the survivorships free of revision were similar in both groups. Concerning the interval time between arthroscopy and TKA, Anthony found patients who had a knee arthroscopy within 1 year to receive TKA were not having a higher risk of complications or reoperations. Werner and Barton et al. [8, 10] also reported there were not increasing in complications when TKA patient had an arthroscopy for more than 6 months, but patients undergoing TKA within 6 months of arthroscopy had a significantly higher rate of complications and reoperation. In contrast, Piedade [7] reported a higher postoperative complication rate in the arthroscopy group with a mean interval of 53 months.

There are also limitations to our study. Foremost, this is a single-center clinical trial containing only 56 patients. Analysis of the impact of interval time between arthroscopy and TKA was not performed. Some documents have shown that the interval time between arthroscopy and TKA is a potential factor which may influence complication rates and clinical outcomes. Moreover, our study has the common shortcomings, in any retrospective cohort study, of including the possibility of selection or observational bias. Despite these limitations, we pioneered to conduct this comparison on the same patient, the results we have given are still credible.

## Conclusions

For patients who have previously received arthroscopic knee surgery, it is safe to perform TKA, if the illness condition requires it, and the postoperative complications and recovery of knee function risks are the same as in common patients.

## Data Availability

The datasets generated during and/or analyzed during the current study are available from the corresponding author on reasonable request.

## Abbreviations

TKA: Total knee total knee arthroplasty
ROM: Range of motion
FJS: Finger joint size
VAS: Visual analogue scale
VTE: Venous thromboembolism
BMI: Body mass index
ASA: American society of anesthesiologists
KSS: Keen society score
UKA: Unicompartmental knee arthroplasty
MP: Medial pivot
PS: Posterior-Stabilized
IKS: International Knee Society
